# Programmed death-1in cancer stem cells may serve as a novel immunotherapy marker

**DOI:** 10.1186/1878-5085-5-S1-A115

**Published:** 2014-02-11

**Authors:** Babak Baban, Jun Yao Liu, Mei Zheng, Jeffrey Lee, Karolina Grochowska, Mahmood Mozaffari

**Affiliations:** 1 Department of Oral Biology, College of Dental Medicine/ School of Medicine, Georgia Regents University, Augusta, Georgia 30912, USA; 2 Department of Pathology, College of Dental Medicine/ School of Medicine, Georgia Regents University, Augusta, Georgia 30912, USA

## Scientific objective

Head and neck cancers (HNC) are among the most prevalent and deadly cancers. Despite advances in treatment and improvement in patient’s quality of life, survival rates have not improved in these patients. Increasing evidence shows that the initiation, growth and metastasis of cancer are directed by a small functional subpopulation of cancer cells defined as cancer stem cells (CSCc). Multipotency and differentiation into the tumor cells, plasticity and self-renewal are among the most exclusive features of CSCs. Programmed death 1 (PD-1) protein, is an inhibitory T cell receptor which is able to muffle the immune response of activated T cells, helping cancer cells to evade the host defense system. Thus, we have examined the presence of CSCs in HNCs and if there is any correlation between HNC development and PD-1 expression.

## Technical approach/methods

Using tissue microarrays, we employed immunohistochemistry and immunofluorescent protocols to explore if CSCs marker, CD44, is expressed within the tumor microenvironment. In addition, we investigated the presence of PD-1 expressing cells as inhibitors of antitumor immunity.

## Results/interpretation

We observed that a subpopulation of HNC expressed CD44 which is suggestive of the presence of CSCs within the tumor. Moreover, PD-1 was expressed within the tumor microenvironment thereby indicating the pivotal role of PD-1 in the suppression of the host immune system and its significance for tumor survival and progression of cancer. Collectively, the results not only re-emphasize the role of CSCs in initiation and establishment of HNC, but also suggest PD-1 as a novel therapeutic target in anticancer frontier.

## Outlook/expert recommendations

The present study suggests, for the first time, a relation between cancer stem cells and programmed cell death 1 in head and neck cancer. Our findings indicate that the presence of cancer stem cells in the head and neck tumor and their association with the tumor structure may play a crucial role not only in the establishment of the tumor, but also, in the induction of immunosuppression status of the immune system against head and neck tumors. Therefore, subsequent studies should determine whether any alteration in the presence and activity of cancer stem cells within the tumor microenvironment can up-regulate the immune responses against tumor growth and progression in the head and neck cancer. The primary therapeutic impact of such stimulation of immune system will results in the focal disruptions of the tumor capsule, which selectively favor tumor stem cells proliferation and invasion.

